# iPSC-derived progenitor stromal cells provide new insights into aberrant musculoskeletal development and resistance to cancer in down syndrome

**DOI:** 10.1038/s41598-020-69418-9

**Published:** 2020-08-06

**Authors:** Yekaterina Galat, Mariana Perepitchka, Irina Elcheva, Stephen Iannaccone, Philip M. Iannaccone, Vasiliy Galat

**Affiliations:** 1 Developmental Biology Program, Stanley Manne Children’s Research Institute, Ann & Robert H. Lurie Children’s Hospital, Chicago, IL USA; 2 Pediatrics, Northwestern University Feinberg School of Medicine, Chicago, IL USA; 3 Pathology, Northwestern University Feinberg School of Medicine, Chicago, IL USA; 4 Robert H. Lurie Comprehensive Cancer Center, Northwestern University Feinberg School of Medicine, Chicago, IL USA; 5 ARTEC Biotech Inc, Chicago, IL USA; 6 Present Address: Pediatrics, Division of Hematology and Oncology, Penn State Hershey College of Medicine, Hershey, PA USA

**Keywords:** Cancer microenvironment, Cellular signalling networks, Disease model, Induced pluripotent stem cells

## Abstract

Down syndrome (DS) is a congenital disorder caused by trisomy 21 (T21). It is associated with cognitive impairment, muscle hypotonia, heart defects, and other clinical anomalies. At the same time, individuals with Down syndrome have lower prevalence of solid tumor formation. To gain new insights into aberrant DS development during early stages of mesoderm formation and its possible connection to lower solid tumor prevalence, we developed the first model of two types of DS iPSC-derived stromal cells. Utilizing bioinformatic and functional analyses, we identified over 100 genes with coordinated expression among mesodermal and endothelial cell types. The most significantly down-regulated processes in DS mesodermal progenitors were associated with decreased stromal progenitor performance related to connective tissue organization as well as muscle development and functionality. The differentially expressed genes included cytoskeleton-related genes (actin and myosin), ECM genes (Collagens, Galectin-1, Fibronectin, Heparan Sulfate, LOX, FAK1), cell cycle genes (USP16, S1P complexes), and DNA damage repair genes. For DS endothelial cells, our analysis revealed most down-regulated genes associated with cellular response to external stimuli, cell migration, and immune response (inflammation-based). Together with functional assays, these results suggest an impairment in mesodermal development capacity during early stages, which likely translates into connective tissue impairment in DS patients. We further determined that, despite differences in functional processes and characteristics, a significant number of differentially regulated genes involved in tumorigenesis were expressed in a highly coordinated manner across endothelial and mesodermal cells. These findings strongly suggest that microRNAs (miR-24-4, miR-21), cytoskeleton remodeling, response to stimuli, and inflammation can impact resistance to tumorigenesis in DS patients. Furthermore, we also show that endothelial cell functionality is impaired, and when combined with angiogenic inhibition, it can provide another mechanism for decreased solid tumor development. We propose that the same processes, which specify the basis of connective tissue impairment observed in DS patients, potentially impart a resistance to cancer by hindering tumor progression and metastasis. We further establish that cancer-related genes on Chromosome 21 are up-regulated, while genome-wide cancer-related genes are down-regulated. These results suggest that trisomy 21 induces a modified regulation and compensation of many biochemical pathways across the genome. Such downstream interactions may contribute toward promoting tumor resistant mechanisms.

## Introduction

Down syndrome (DS) is a complex disease caused by a trisomy of human chromosome 21 (HSA21) that occurs at a rate of 1 in every 750 births. Children with DS show a spectrum of clinical anomalies including cognitive impairment, musculoskeletal and blood disorders, cardiac malformations, and others. At the same time, they have a reduced incidence of solid tumors^1^.

We and others have shown that differentiated cells, produced from human pluripotent stem cells (hPSCs), recapitulate major embryonic developmental steps and can be employed as experimental models in the study of human diseases^2–4^. To date, the majority of DS hPSC studies addressed neural development, but there are also several investigations, involving the “mesodermal compartment”, that are dedicated to hematopoiesis. One current hypothesis is that T21 increases hematopoiesis and leads to a disbalance in cell fate and proliferation of particular hematopoietic lineages^5–8^, which compliments the existing explanation that acquired GATA1 mutations drive leukemia incidences in DS patients^9,10^.

Based on our previous experience with mesodermal and endothelial differentiation of pluripotent stem cells^11–16^, we investigated a DS iPSC-derived mesodermal and endothelial cellular model. These two types of stromal cells contribute to the mechanical and structural components of the musculoskeletal system. At the same time, they constitute the main component of the solid tumor microenvironment.

DS patients demonstrate an increased risk of numerous cancer factors such as: chromosomal instability, increased DNA damage^17^, inflammatory conditions, immune abnormalities, clinical immunodeficiency^18^, and excessive weight gain. Nevertheless, DS individuals have a low risk of solid tumors^19^, which represents a paradox^20^. Several explanations have been offered proposing that mechanisms of reduced solid tumor incidence are driven by an increased expression of Chromosome 21 tumor suppressor genes, in particular, those leading to angiogenic inhibition^21–25^. It was also suggested that such a tumor resistant role of T21 goes beyond reduced angiogenesis and involves multiple mechanisms that contribute to reduced cancer mortality^26^. Other hypotheses highlighted a possible tumor suppressive role of stromal cells^27^. Specifically, it was noted that leukemic and testicular cancers, which have a higher incidence in DS individuals, are lacking or have poorly developed stroma.

Here, we establish a set of 30 genes on Chromosome 21 and over 100 genome-wide genes showing that T21 induces a modified regulation of many interacting pathways across the genome. Utilizing pathway analyses and functional assays, we show that the top down-regulated processes involve the cytoskeleton, cell cycle, and ECM organization, which reflects an impairment in mesodermal progenitor function. At the same time, the most down-regulated processes in endothelial cells involve a response to external stimuli, cell migration, and immune response (inflammation-based), which also suggests decreased functional performance. These obstacles likely specify the basis of connective tissue impairment in DS patients. When coupled with angiogenic inhibitions, they potentially hinder tumor progression and exhibit a “resistant to solid cancer” phenotype.

## Results

**Figure 1:**
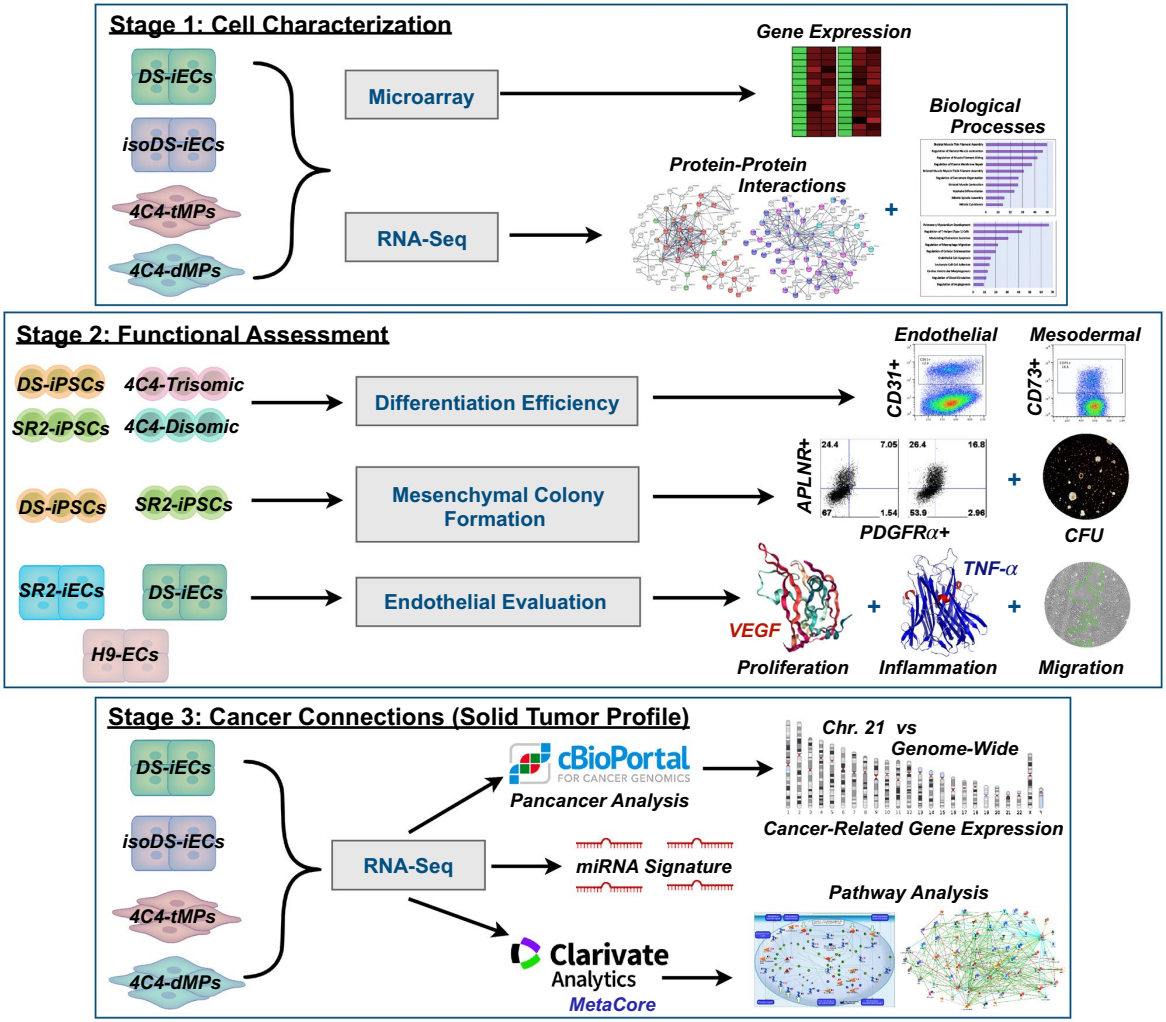
Schematic study design. Outline of three experimental stages: (1) Cell characterization, (2) functional assessment, (3) cancer connections (solid tumor profile). The cell lines, assays, and/or bioinformatic analyses are included for each stage. SR2-iPSCs, DS-iPSCs, 4C4-Disomic, and 4C4-Trisomic are isogenic stem cell lines. 4C4-dMPs and 4C4-tMPs are the disomic and trisomic mesodermal progenitors. H9-ECs, SR2-iECs, DS-iECs, and isoDS-iECs are endothelial cells.

This study is divided into three experimental stages: Cell Characterization (Isogenic Clone Profile), Functional Assessment, and Cancer Connections (Solid Tumor Profile). Each stage is an important contributor toward shaping an in-depth evaluation of the impact of DS on the solid tumor microenvironment (Fig. 1).

## Cell characterization: isogenic clone profile

### Mesodermal progenitor and endothelial cell derivation and characterization

To assess the progenitor stromal contribution to the DS phenotype, we used iPSCs from two pairs of isogenic clones established from two different individuals. Additionally, we included a euploid iPSC line (SR2-iPSCs) and a euploid ESC line (H9-ESCs) for endothelial cell migration assessment. The mesodermal progenitors, 4C4-dMPs and 4C4-tMPs, were derived from isogenic lines 4C4-Disomic and 4C4-Trisomic^8^. Endothelial cells SR2-iECs and H9-ECs were derived from SR2-iPSCs and H9-ESCs. DS-iECs (trisomic isogenic cell line) and isoDS-iECs (disomic isogenic cell line) were both derived from DS-iPSCs^12^. Progenitor cell derivation was performed using a monolayer CHIR99021 induction protocol^13^.

**Figure 2:**
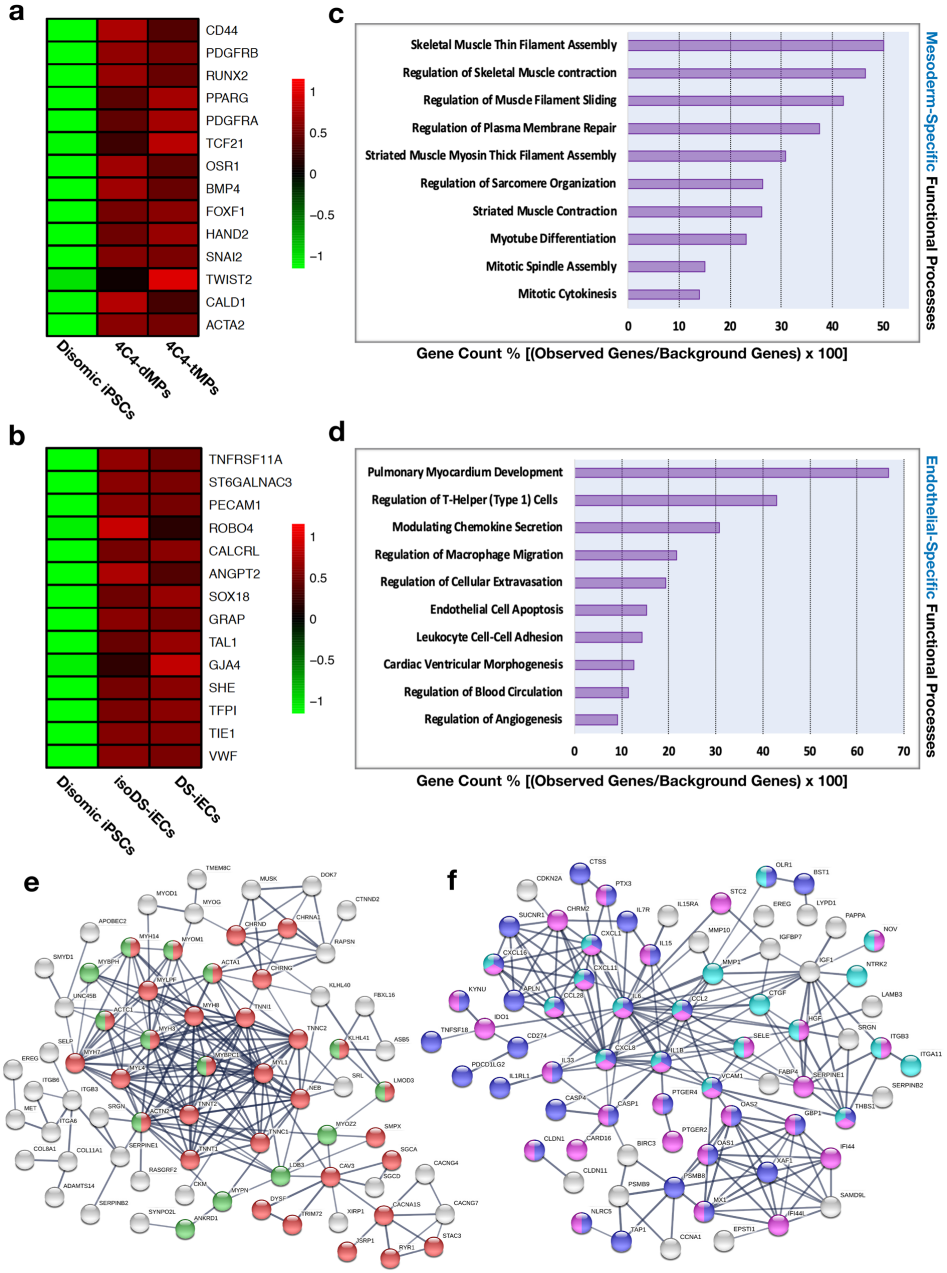
Characterization of progenitor stromal cells. (**a**) Heatmap showing mesoderm-related gene expression correlation among isogenic clones 4C4-dMPs and 4C4-tMPs. The microarray expression data has been log-transformed. (**b**) Heatmap showing endothelium-related gene expression correlation among isogenic clones isoDS-iECs and DS-iECs. The microarray expression data has been log-transformed. (**c**) Statistically significant, mesoderm-specific functional processes identified using STRING database. Observed genes refer to the microarray gene set, and the background genes comprise all genes involved in the biological process. (**d**) Statistically significant, endothelial-specific functional processes identified using STRING database. Observed genes refer to the microarray gene set, and the background genes comprise all genes involved in the biological process. (**e**) Interaction network for 200 most down-regulated genes in 4C4-tMPs, constructed using STRING database (confidence score = 0.700). The gene cluster contains genes related to myogenic development. (**f**) Interaction network for 200 most down-regulated genes in DS-iECs, constructed using STRING database (confidence score = 0.700). The gene cluster contains genes related to stimulus response, migration, and inflammatory response.

Cell characterization was evaluated on the basis of mesodermal and endothelial gene expression and assessment of functional processes. Via R-Studio software, we utilized microarray data to generate heatmaps, which highlight the mesodermal gene expression profile of 4C4-dMPs and 4C4-tMPs (Fig. 2a) as well as the endothelial gene expression profile of DS-iECs and isoDS-iECs (Fig. 2b). Following this, we employed the STRING database (confidence score = 0.700) in order to identify statistically significant, lineage-specific functional correlations. We utilized the top 500 differentially expressed genes in our RNA-Seq dataset for this analysis. The resulting data complements the respective gene expression profiles. 4C4-dMP and 4C4-tMP mesodermal progenitors are predominately associated with muscle development (Fig. 2c), while endothelial cells, DS-iECs and isoDS-iECs, are affiliated with angiogenic, migratory, and inflammatory functionalities (Fig. 2d).

Having confirmed lineage-specificity, we evaluated gene interactions correlating to the identified functional processes. We utilized the STRING database and RNA-Seq gene expression data for this analysis. Interaction networks for 200 most down-regulated genes and 200 most up-regulated genes, with respect to trisomic cells, were obtained for both mesodermal progenitor (4C4-dMP, 4C4-tMP) and endothelial (DS-iEC, isoDS-iEC) cell lines. Out of the three interaction maps (confidence score = 0.700), the most down-regulated mesodermal progenitor and endothelial gene sets produced the most informative interactions. These networks comprised the largest number of protein–protein connectivity among 200 genes.

### Trisomic mesodermal progenitors have down-regulated cytoskeletal gene expression

A more detailed inspection of our mesodermal progenitor gene set highlighted the prevalent role of actin and myosin cytoskeletal filaments. Actin and myosin cytoskeletal filaments contribute to myogenic development by ensuring appropriate muscle tissue organization, which directly affects functionality. For the 4C4-dMP and 4C4-tMP dataset, the STRING database constructed the following gene cluster, which is involved in actomyosin structural organization and muscle contraction: ACTN2, ACTC1, MYBPC1, MYH3, MYH14, MYOM1, ACTA1, KLHL41, and LMOD3 (Fig. 2e).

### Trisomic endothelial cells have a down-regulated expression of genes involved in stimulus response, migration, and immune response (inflammation-based)

Endothelial STRING analysis of the most down-regulated 200 genes in DS-iECs vs. isoDS-iECs revealed several clusters of gene interactions. The most significant processes identified were response to chemical stimuli, cellular migration, and immune response (inflammation-based). The following genes help facilitate all three cellular functions: CXCL16, CXCL1, CXCL11, CCL28, IL6, CXCL8, CCL2, IL1β, VCAM-1, and THBS1. In light of the interconnected involvement of these genes, this data highlights that trisomic gene down-regulation affects endothelial function via internal and external cellular mechanisms (Fig. 2f).

## Functional assessment

### Mesodermal evaluation

#### Differentiation and development of mesodermal progenitor cells

Considering our finding that the most down-regulated processes highlighted impaired muscle system development, we conducted mesodermal differentiation efficiency assays. We used isogenic iPSC lines 4C4-Disomic, 4C4-Trisomic, SR2-iPSCs, and DS-iPSCs to evaluate early stages of mesodermal development.

**Figure 3:**
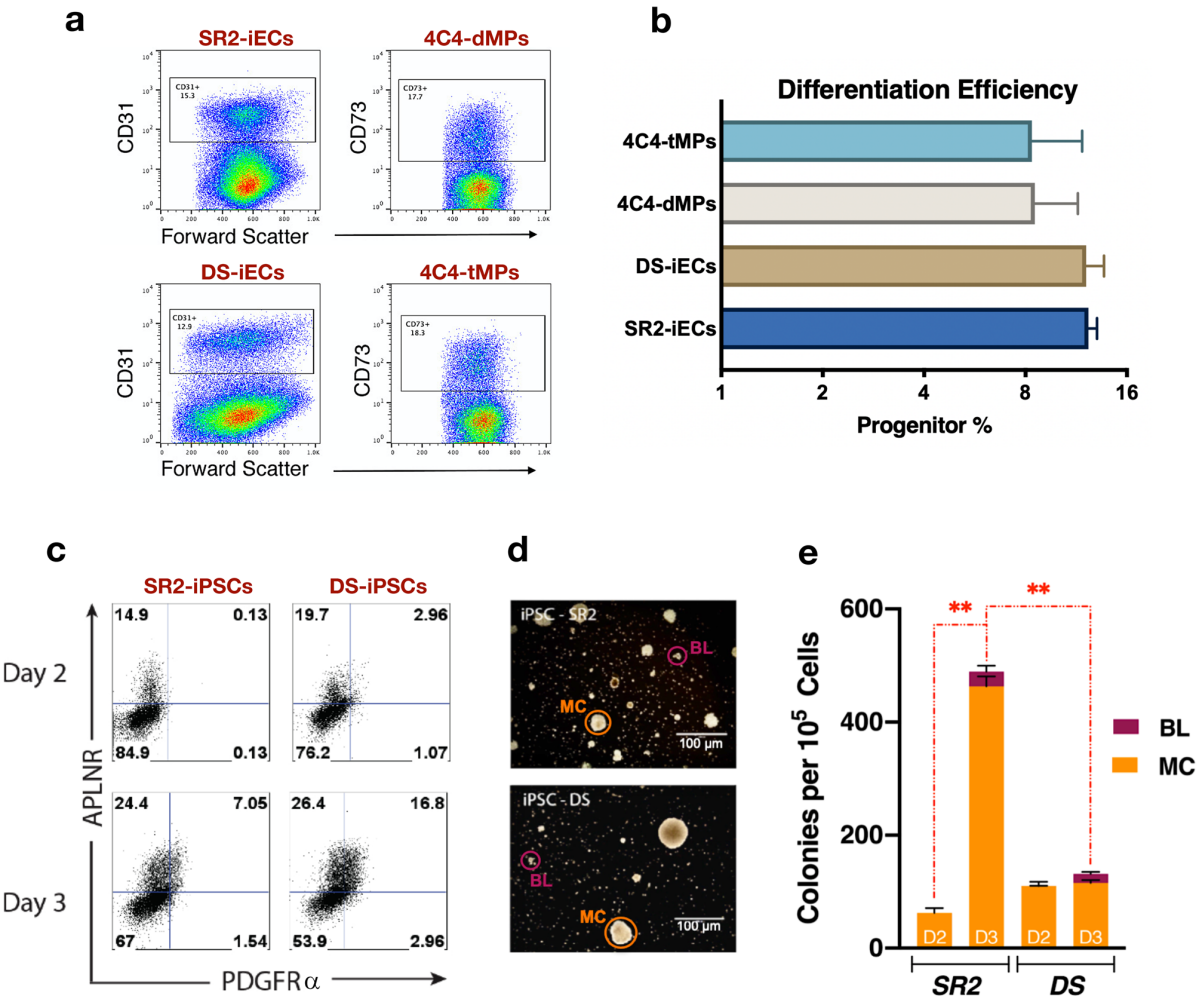
Assessment of stromal progenitor differentiation efficiency and ds mesodermal progenitor function. (**a**) Representative images of flow cytometry results of (i) DS-iEC and SR2-iEC differentiation efficiency. The efficiency is assessed by the percentage of CD31 + cells; (ii) 4C4-dMP and 4C4-tMP differentiation efficiency. The efficiency is assessed by the percentage of CD73 + cells. (**b**) Differentiation efficiency of mesodermal and endothelial progenitors, evaluated using the monolayer induction protocol. (**c**) Representative images of flow cytometry results of SR2- and DS-derived progenitors on day 2 and day 3 of differentiation using an OP9 co-culture protocol. APLNR + PDGFRα + cells represent the mesenchymoangioblast population. (**d**) Representative phase contrast microscopy images of Blast (BL) and Mesenchymal (MC) colonies formed after SR2 and DS mesenchymoangioblasts were isolated on day 3 and cultured for 16 days in semi-solid media. (**e**) Graph showing that SR2 disomic progenitors isolated on day 3 of differentiation formed a significantly (pV < 0.0001) greater amount of MC colonies compared to the DS trisomic progenitors. Error bar represents SEM from three independent experiments.

First, using the CHIR99021 induction protocol, we compared mesodermal and endothelial differentiation efficiency of two sets of trisomic and disomic isogenic cell lines. The results were assessed by flow cytometric analysis. The number of endothelial progenitors (SR2-iECs and DS-iECs) was documented with the CD31 + cell population, while mesodermal progenitors (4C4-dMPs and 4C4-tMPs) comprised the CD73 + fraction (Fig. 3a). The average percent of CD73 + cells for 4C4-Disomic was 8.5% and 8.4% for 4C4-Trisomic. The average percent for CD31 + cells for SR2-iPSCs and DS-iPSCs was 12.3% and 12.1%, respectively. We found no significant difference between the percentages of mesodermal and endothelial cells generated at day 5 of disomic vs. trisomic iPSC differentiation (Fig. 3b).

#### DS mesodermal progenitors form less mesenchymal colonies in comparison to disomic mesodermal progenitors

To further explore the reason behind impaired mesodermal formation, we paired another differentiation protocol with a CFU assay to evaluate the proliferative capacity of the derived mesenchymal precursors (mesenchymoangioblasts). Mesenchymoangioblasts are characterized by APLNR^+^PDGFRα^+^KDR^+^ and were shown to give rise to mesenchymal and endothelial cells^28^. Therefore, we first evaluated RNA-Seq expression of APLNR, PDGFRα, and KDR genes. We found that PDGFRα expression was up-regulated in both MPs (fold change (FC) = 1.72, *p *values (pV) < 0.0001) and iECs (FC = 1.36, pV = 2.07 × 10^−15^). KDR expression was also up-regulated in MPs (FC = 4.74, pV = < 0.0001) and in iECs (FC = 1.03, pV < 0.0001). APLNR expression, on the other hand, was not part of the mesodermal RNA-Seq dataset, but it was up-regulated in iECs (FC = 6.75, pV < 0.0001). This expression profile suggested that DS cells should have an increased mesenchymoangioblast differentiation potential.

To confirm this finding, we used the OP9 mouse stromal cell co-culture method for mesenchymoangioblast differentiation. DS and SR2 iPSCs were plated on an overconfluent layer of OP9 cells and collected on day 2 and day 3 of differentiation. The collected progenitors were evaluated for the expression of APLNR and PDGFRα by flow cytometry and then placed in MethoCult media for CFU analysis. Flow cytometric analysis verified the RNA-Seq data and showed that the DS differentiating culture contained a higher percentage of APLNR and PDGFRα expressing progenitors on both day 2 and day 3 of differentiation (Fig. 3c). Interestingly, when placed into semi-solid media and allowed to grow for 16 days, DS progenitors formed significantly less MS colonies than the SR2 disomic cultures (Fig. 3d,e).

### Endothelial evaluation

#### DS-iECs have a decreased response to VEGF

**Figure 4:**
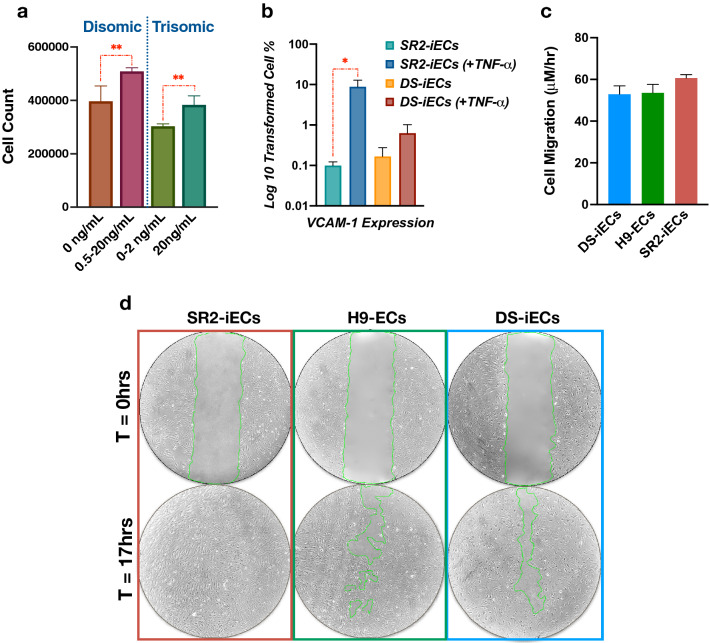
Functional assessment of endothelial progenitors. (**a**) Disomic and Trisomic cell counts following 0.5 ng/mL, 2 ng/mL, and 20 ng/mL VEGF addition. Error bars represent SEM from three replicates. (**b**) Inflammatory Assay data based on iEC response to TNF-α stimulation. The sensitivity of the response was evaluated on the basis of VCAM-1. Error bar represents SEM incorporating four experimental replicates. (**c**) A graph demonstrating the cell migration rate of DS-iECs, H9-ECs and SR2-iECs. There was no significant difference in migration rate between the cell lines. (**d**) Representative phase contrast microscopy images of the scratch assay comparing migration rates of DS-iECs, H9-ECs and SR2-iECs.

To substantiate our finding that DS-iECs have a down-regulated response to stimuli, we investigated the effect of VEGF addition on cellular proliferation. We chose VEGF concentrations of 0.5 ng/mL, 2 ng/mL, and 20 ng/mL on the basis of free VEGF secretions^29^. We then measured the rate of cellular proliferation in response to these different VEGF concentrations. In this assay, we compared trisomic and disomic iEC lines. We found that DS-iECs were less proliferative and had a diminished response to VEGF in comparison to disomic controls. At a concentration of 0.5 ng/mL VEGF, there was a significant increase in disomic cell proliferation, but DS-iECs had no significant response. The rate of DS-iEC proliferation was relatively the same at 0, 0.5, and 2 ng/mL concentrations. With the addition of 20 ng/mL VEGF (10 × the dose of VEGF required to elicit a response in disomic cells), the rate of DS-iEC proliferation significantly increased (Fig. 4a).

#### DS-iECs exhibit less sensitivity to TNF-α stimulation

To functionally evaluate endothelial immune response, we performed an inflammatory assay utilizing trisomic endothelial cells (DS-iECs) and disomic endothelial cells (SR2-iECs). We selected endothelial cells for this analysis because they had a more significant down-regulatory gene expression trend in comparison to trisomic mesodermal progenitors (4C4-tMPs). Since intercellular adhesion molecule-1 has been used as an endothelial drug delivery site for inflammatory therapies, we decided to measure inflammatory response relative to this molecule^30^. More specifically, we decided to focus on VCAM-1 expression because, in comparison to ICAM-1, VCAM-1 had a larger fold change in expression (5.102 > 3.918). Our results showed that, after TNF-α treatment, SR2-iECs exhibited a significant increase in VCAM-1 expression while DS-iECs demonstrated no significant difference in response (Fig. 4b). These functional results align with the down-regulated inflammatory gene expression trend (Table 1).

**Table 1:** Inflammatory gene expression profile.

Inflammatory		Endothelial		Mesodermal		
Genes	DS-iECs	isoDS-iECs	*p* values	4C4-tMPs	4C4-dMPs	*p* values
*ADRA2A*	79.75	**5.42**	5.09E−14	**0.00**	71.76	3.71E−17
*AIM2*	44.54	**4.81**	8.50E−08	18.50	**2.63**	0.0011
*APOE*	656.52	**48.27**	2.40E−88	19,188.30	**3,396.90**	1.80E−201
*C3AR1*	**10.14**	64.24	3.02E−09	**48.98**	165.80	4.39E−13
*CCL2*	**38.14**	4,901.26	< 0.0001	43.74	**1.91**	9.32E−10
*CD44*	**2,985.34**	23,055.09	< 0.0001	**6,621.68**	32,337.04	< 0.0001
*CLU*	**530.05**	4,370.24	< 0.0001	**3,378.93**	11,478.69	< 0.0001
*EDNRA*	29.74	130.20	1.16E−13	2,103.29	**480.81**	4.28E−141
*EDNRB*	170.55	**33.65**	1.53E−18	4,657.29	**1,165.44**	7.27E−271
*EPHB6*	329.11	**51.38**	3.05E−38	50.50	**7.16**	5.88E−08
*FABP4*	**13.86**	420.10	6.26E−65	**5.52**	40.00	0.0120
*FANCD2*	1924.36	**301.17**	9.50E−198	731.15	**108.78**	1.07E−83
*HMGB2*	8,113.09	**1,262.47**	< 0.0001	2,725.25	**453.16**	2.96E−251
*ICAM-1*	**1,041.73**	15,773.19	< 0.0001	**1894.50**	6,940.98	< 0.0001
*IL17B*	21.47	4.00	0.0012	**39.28**	119.40	2.02E−08
*IL1A*	**61.21**	597.47	8.31E−85	95.45	**23.88**	1.91E−09
*IL1B*	**14.13**	393.84	1.29E−61	136.62	**30.99**	7.02E−14
*IL6*	**0.53**	271.73	9.12E−32	**2.43**	19.16	0.0005
*ITGA2*	**1,331.36**	7,214.49	< 0.0001	**476.07**	2059.10	2.87E−152
*MGLL*	**184.96**	1,240.48	1.82E−138	**95.61**	602.00	2.61E−68
*OLR1*	**0.53**	65.27	1.47E−14	**380.53**	1873.28	5.42E−148
*P2RX7*	**15.73**	113.67	1.42E−16	61.53	**15.94**	6.79E−06
*PTGER4*	**6.80**	295.08	7.39E−46	535.60	**92.30**	1.16E−57
*PTGS2*	**130.82**	612.76	1.89E−51	**69.45**	241.98	8.45E−19
*SELP*	**333.92**	1528.60	1.43E−121	**0.53**	39.70	1.06E−10
*SERPINE1*	**698.87**	147,811.18	< 0.0001	**2,103.56**	81,571.22	< 0.0001
*SUCNR1*	**2.13**	135.01	5.72E−24	**8.18**	130.58	1.39E−22
*SYK*	43.34	**6.50**	8.75E−07	835.51	**20.18**	3.76E−108
*TNFSF18*	**2.94**	786.18	5.17E−67	**26.24**	1,010.74	1.57E−135
*TNFSF4*	**104.02**	1834.75	5.11E−264	**2,221.03**	6,918.11	2.43E−260

Top 30 statistically significant, inflammatory genes (fold change > 1.5) across mesodermal progenitors (4C4-dMPs, 4C4-tMPs) and endothelial cells (DS-iECs, isoDS-iECs). Both trisomic mesodermal and endothelial cells exhibit a down-regulatory inflammatory gene expression profile, as indicated by the bold numbers.

#### DS-iECs have comparable migratory rates to disomic iECs

We further evaluated the vasculogenic potential of DS-iECs, compared to control SR2-iECs and H9-ECs, via the migration assay. The width of the scratch area across all cell lines ranged from 705 to 714.39 μM. After 17hrs, SR2-iECs reached full confluency, and the migration rate was calculated. The average rate of cell migration for DS-iECs was 49.98 μM/h. H9-ECs and SR2-iECs displayed migratory rates of 56.76 μM/h and 60.40 μM/h. Statistical analysis did not reveal a significant difference in the migration rates (Fig. 4c,d). This indicates that mis-regulation of cellular motility may not factor into the DS phenotype.

## Cancer connections: solid tumor profile

### Building a cancer profile for down syndrome

**Figure 5:**
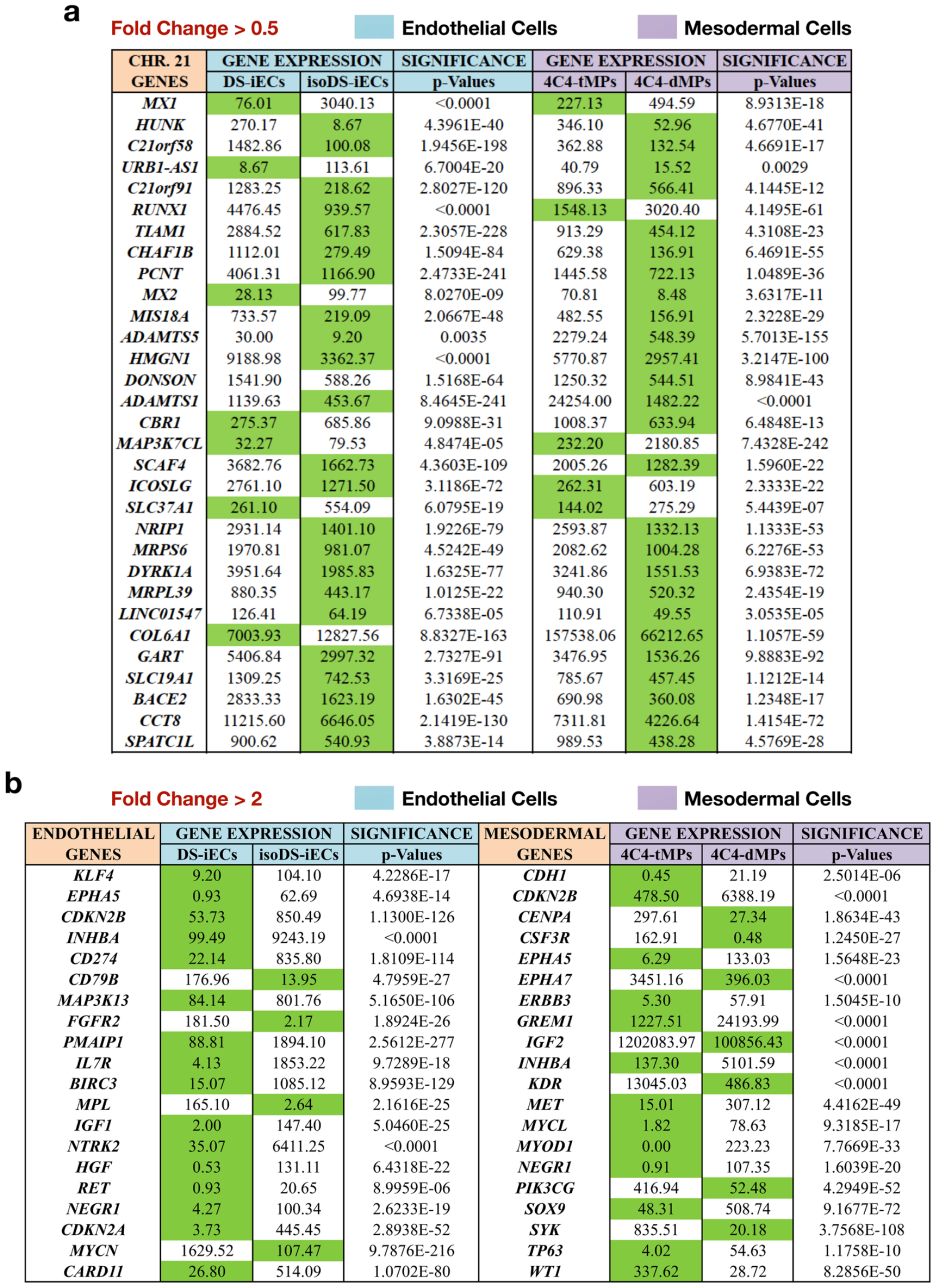
Chromosome 21 and genome-wide cancer-related gene expression profiles. (**a**) List of the top 30 statistically significant cancer-related genes specific to Chromosome 21 (fold change > 0.5). Trisomic mesodermal progenitors and endothelial cells show an up-regulatory expression trend. (**b**) List of the top 20 statistically significant genome-wide cancer-related solid tumor genes (fold change > 2). Trisomic mesodermal progenitors and endothelial cells exhibit a down-regulatory expression trend vs the disomic controls (4C4-dMPs and isoDS-iECs). In both gene tables, the green color designates down-regulated gene expression.

To investigate the genetic implications of DS on cancer development, we identified cancer-related genes expressed on Chromosome 21. Our approach involved utilizing the Cancer Genome Atlas (cBioPortal)^31,32^ to select a thorough pancancer analysis incorporating studies of 35 cancer types (liquid and solid tumors). The collected data was obtained from 11,000 patients. Furthermore, this analysis provided a list of the most frequently mutated genes across cancer cases. We used our RNA-Seq data to locate which Chromosome 21-specific genes from our mesodermal progenitors (4C4-dMPs, 4C4-tMPs) and endothelial cells (DS-iECs, isoDS-iECs) appeared on the list obtained from the Cancer Genome Atlas. We identified 30 genes: MX1, HUNK, C21orf58, URB1-AS1, C21orf91, RUNX1, TIAM1, CHAF1B, PCNT, MX2, MIS18A, ADAMTS5, HMGN1, DONSON, ADAMST1, CBR1, MAP3K7CL, SCAF4, ICOSLG, SLC37A1, NRIP1, MRPS6, DYRK1A, MRPL39, LINC01547, COL6A1, GART, SLC19A1, BACE2, CCT8, and SPATC1L. In addition to this, we also noticed a consistent trend of significant gene up-regulation. In trisomic mesodermal progenitors (4C4-tMPs), 25 out of 30 genes were up-regulated; in trisomic endothelial cells (DS-iECs), 23 out of 30 genes were up-regulated (Fig. 5a).

### Down-regulatory impact of down syndrome on genome-wide cancer-related gene expression

Following our study of Chromosome 21 cancer-related genes, we evaluated the impact of DS on cancer development from a genome-wide perspective. Similarly to the previous analysis, we utilized the Cancer Genome Atlas (cBioPortal) pancancer analysis incorporating 35 cancer studies. We then selected the top 10 cancer cases affecting the largest number of patients across a total of 11,000 patients: invasive breast carcinoma, non-small cell lung cancer, colorectal adenocarcinoma, glioblastoma, endometrial carcinoma, ovarian epithelial tumor, head and neck squamous cell carcinoma, esophagogastric adenocarcinoma, diffuse glioma, and renal clear cell carcinoma. Following that, we obtained a gene list containing the most frequently mutated genes across these 10 cancer cases, which involve the solid tumor microenvironment. A comparison of this gene list with our mesodermal and endothelial RNA-Seq data revealed significantly expressed genes for each cell type. In contrast to the Chromosome 21 cancer-related genes that showed an up-regulated expression trend, both trisomic mesodermal progenitors (4C4-tMPs) and endothelial cells (DS-iECs) exhibited a down-regulatory expression trend vs. disomic 4C4-dMPs and isoDS-iECs (Fig. 5b).

### miRNA tumor signature in down syndrome

In order to further develop our DS cancer profile, we evaluated miRNA expression levels since miRNAs can function both as oncogenes and tumor suppressors. Previous research has also shown that miRNA expression is correlated with cancer-specific signatures^33^. We targeted miRNAs that are commonly up-regulated in two or more solid tumor types. Our RNA-Seq data revealed eight significantly expressed miRNAs. miR-146a, miR-20, miR-25, and miR-221 were part of the endothelial dataset (DS-iECs vs. isoDS-iECs); miR-24-1 and miR-92-2 were present in the mesodermal dataset (4C4-dMPs vs. 4C4-tMPs). Both mesodermal progenitors and endothelial cells expressed miR-21 and miR-24-2. miR-21 shares a signature with six solid tumor types: breast, colon, lung, pancreas, prostate, and stomach; miR-24-2 signature is affiliated with colon, pancreas, and stomach tumors^34,35^. Both miR-21 and miR-24-2 expression levels, which are normally up-regulated in these solid tumors, are instead significantly down-regulated (pV < 0.0001) in our trisomic 4C4-tMPs and DS-iECs.

### Down syndrome MPs and iECs show signs of increased gene expression linked to cell cycle regulation

Additionally, we evaluated mechanisms that contribute to cancer and myogenic development. Namely, cell proliferative capacity, which can be accessed via cell cycle checkpoint pathways that regulate DNA damage repair mechanisms. USP16 is associated with DNA damage accumulation in the cells. In our DS model, USP16 is up-regulated in both mesodermal (FC = 0.87, pV = 7.03 × 10^−43^) and endothelial (FC = 0.31, pV = 3.00 × 10^−6^) progenitor cells.

Furthermore, our MetaCore enrichment analysis of RNA-Seq data showed that the top 10 statistically significant functional process networks revolve around cell cycle phases and DNA damage response (Supplemental Figure S1a). A deeper analysis of the ATM and ATR kinase activation pathways was done because they incorporate multiple DNA repair mechanisms (Supplemental Figure S1b). Interestingly, out of the 23 genes that are part of our mesodermal RNA-Seq dataset, only 2 genes are down-regulated, and the remaining 21 genes are up-regulated. These results also correlate well with our DS endothelial gene expression profile. For the same pathway, out of the 24 genes present in our RNA-Seq endothelial dataset, 2 genes are down-regulated and 22 genes are up-regulated. This correlation highlights the possibility that DS widespread chromosomal dysregulation impacts cell cycle progression and DNA damage response, regardless of cell type.

In terms of cell type-specific pathway regulation, the key pathways for endothelial cell function involved down-regulation of the HIF-1 complex (Supplemental Figure S2a), while key pathways for mesodermal myogenic processes involved up-regulation of the S1P receptor complex (Supplemental Figure S2b).

### Down-regulation of ECM component expression in down syndrome

The extracellular matrix (ECM) is a complex, versatile structure that undergoes constant remodeling. Dysregulation of the ECM is highly involved in tumor development and progression^36,37^. Type IV Collagen, Galectin-1, proteoglycans, and glycoproteins are major components of the ECM. We evaluated our data set with regard to ECM component expression in DS cells compared to the disomic controls. The data revealed that key components of the ECM were down-regulated in both sets of isogenic DS progenitors:

Galectin-1, a protein that facilitates adhesion to the ECM, increased migration, and stromal immune suppression, was down-regulated in 4C4-tMPs (FC = − 0.57, pV = 1.61 × 10^−52^) and in DS-iECs (FC = − 2.57, pV < 0.0001).

Heparan Sulfate (HSPG2), a proteoglycan that helps maintain the physical connections between different ECM components, is associated with the following down-regulated expression values: 4C4-tMPs (FC = − 1.37, pV = 1.28 × 10^−237^) and DS-iECs (FC = − 1.68, pV = 8.06 × 10^−209^).

Fibronectin-1, a ligand for β-integrins that mediate cell-ECM signaling, was also down-regulated in DS clones compared to the disomic cell lines. In contrast, Fibronectin is up-regulated in solid tumors and is involved in tumor cell proliferation^38^. With regard to our isogenic DS clones, its down-regulatory expression was affiliated with the following data: 4C4-tMPs (FC = − 2.18, pV < 0.0001) and DS-iECs (FC = − 1.67, pV < 0.0001).

Collagen serves as a scaffold for the tumor microenvironment and affects tumor growth by regulating ECM remodeling via collagen structural modifications, which promote tumor infiltration, angiogenesis, invasion, and migration^39^. Even though our data demonstrate a significant up-regulation in Collagen II, III, VI, XV, and XVI gene expression, we observed a significant down-regulation in Collagen I: (4C4-tMPs, COL1A1, FC = − 0.25, pV = 5.67 × 10^−12^), (DS-iECs, COL1A1, FC = − 1.1, pV = 3.72 × 10^−231^) and Collagen IV: (4C4-tMPs, COL4A1, FC = − 2.64, pV = 0; COL4A2, FC = − 1.73, pV < 0.0001), (DS-iECs, COL4A1, FC = − 0.86, pV = 0; COL4A2, FC = − 0.69, pV = 9.63 × 10^−49^). This collagen expression variability suggests the possibility that the DS genotype creates an internal bodily environment that limits tumor migratory potential.

To further corroborate this proposition, we incorporated a mechanistic perspective into our analysis. Several research studies have targeted lysyl oxidase (LOX) and focal adhesion kinase (FAK1) as key agents in promoting a favorable microenvironment for solid tumor progression via Collagen I and IV interactivity. LOX-mediated Collagen IV cross-linking leads to ECM deposition and tissue stiffness, which drives malignant tumor progression. When spread to target organs, LOX can crosslink Collagen IV and I to remodel the ECM and recruit bone marrow-derived cells to form the pre-metastatic niche^39^. Focal Adhesion Kinase (FAK1), on the other hand, is a cytoplasmic protein tyrosine kinase, which controls cell movement, invasion, survival, gene expression, and cancer stem cell self-renewal^40^. Collagen I enhances the tumor microenvironment activation of FAK1^41^, and currently, FAK1 is one of the major targets for cancer therapy^42^. We observed a significant down-regulation of LOX and FAK1 genes in our 4C4-tMP and DS-iEC progenitors (Supplementary Figure S3).

### Inflammatory gene profile in down syndrome

Inflammatory response can present itself as a combination of three phenotypes: hypoxic, leukocytic, and angiogenic. To ensure survival, cancer cells can adopt any of these inflammatory phenotypes to create a complex metabolic microenvironment, which promotes cell invasion and metastatic growth^43^. To gain insight into the genetic implications governing inflammatory response in our mesodermal progenitors (4C4-dMPs, 4C4-tMPs) and endothelial cells (DS-iECs, isoDS-iECs), we utilized the Gene Ontology database (GO: 0006954)^44, 45^ to compile a list of 691 inflammatory genes. We compared this list with our RNA-Seq data and identified over a hundred differentially expressed genes among all four cell lines. We focused on the top 30 differentially expressed genes with FC > 1.5 (Table 1). The mesodermal progenitors showed a relatively similar distribution of up- (13 genes) and down-regulation (17 genes) in trisomic 4C4-tMPs. For trisomic endothelial DS-iECs, the trend was more significant. 10 genes were up-regulated and 20 genes were down-regulated. Overall, both trisomic mesodermal and endothelial cells exhibit a down-regulatory inflammatory gene expression profile, which may indicate a decreased response to inflammatory stimuli.

## Discussion

DS is characterized by various anomalies associated with stromal progenitor development, including heart and musculoskeletal disorders. Taking into consideration this stromal dysfunction and a work by Costa^46^ indicating the impairment of DS endothelial progenitors, we evaluated the functional and genetic characteristics of our iPSC-derived endothelial and mesodermal cells. Developmentally, as indicated by mesodermal differentiation efficiency experiments, there was no significant difference between the formation of DS and disomic progenitors. Genetically, however, the derived progenitors exhibited signatures indicative of impaired function. As indicated by STRING analysis, most down-regulated processes of mesodermal progenitors were related to muscle development. Additional inspection of the complete gene set revealed that myosin-related genes comprised the majority of the top 20 most down-regulated and statistically significant genes (Table 2). Similarly to myosin, the top 20 actin-related genes also exhibited significant down-regulation (Table 3).

**Table 2:** Myosin-related genes.

Gene List	Log2 FC	*p *values
*ACTC1*	10.35	5.95E−272
*MYL1*	8.78	2.56E−76
*MYBPH*	8.66	1.03 × 10^–103^
*MYH3*	8.26	< 0.0001
*MYOG*	8.09	2.37E−61
*CHRNA1*	7.57	2.35E−130
*CDH6*	7.28	3.75E−297
*TNNT1*	7.20	3.04E−105
*MYLPF*	7.16	7.44E−114
*MYH7*	7.15	3.81E−44
*MYBPC1*	7.05	1.16E−42
*SERPINB7*	6.89	2.63E−171
*ASB5*	6.81	8.21E−39
*TNNI1*	6.73	5.46E−212
*TRIML2*	6.73	2.08E−63
*XIRP1*	6.71	1.59E−41
*MYH8*	6.66	1.00E−36
*UNC45B*	6.58	1.64E−35
*RYR1*	6.57	3.23E−84
*CACNA1S*	6.53	9.79E−39

Myosin-related genes are part of the 20 most down-regulated and statistically significant genes within the mesodermal progenitor RNA-Seq dataset.

**Table 3:** Actin-related genes.

Gene List	Log2 FC	*p *values
*ACTC1*	10.35	5.95E−272
*XIRP1*	6.71	1.59E−41
*ACTA1*	6.42	1.94E−52
*ACTN2*	5.19	1.5E−98
*XIRP2*	4.59	5.76E−16
*AFAP1L1*	3.50	6.53E−30
*ACTBL2*	2.63	5.76E−10
*ABLIM3*	1.85	9.87E−170
*ACTN1*	1.62	< 0.0001
*FSCN2*	1.43	4.01E−3
*ACTG2*	1.20	7.34E−132
*DSTN*	1.13	1.91E-208
*MACF1*	1.01	8.76E-173
*COTL1*	1.01	1.26 × 10–135
*CTTN*	0.99	2.68E−166
*ARPC1B*	0.93	2.08 × 10^–38^
*ACTN4*	0.90	6.82E−115
*ABLIM1*	0.90	1.83E−32
*ACTR3*	0.84	1.61E−98
*CAPG*	0.79	1.25E−20

Mesodermal RNA-Seq data showing the top 20 actin-related genes that exhibit significant down-regulation in expression.

We also observed a significant down-regulation of Endothelin 1 (EDN1) and Ryanodine receptor 1 (RYR1), which is associated with congenital myopathies^47,48^. Within our dataset, this was one of the most down-regulated genes in DS mesodermal progenitor cells (FC = − 6.57, pV = 3.23 × 10^−84^). EDN1, on the other hand, is associated with development of cardiac dysfunction. In vitro studies have shown that it provokes both inotropic and hypertrophic myocardium^49^. Within our data set, EDN1 is down-regulated in DS mesodermal progenitor cells (FC = − 2.59, pV = 4.63 × 10^−15^) and in DS endothelial cells (FC = − 3.86, pV < 0.0001).

Given these results, we explored our data set with regard to other genes that are involved in skeletal and cardiac muscle development and functionality. From a developmental standpoint, previous studies have shown that the collagen matrix is an important factor in stimulating myogenesis. Collagen VI, in particular, promotes the stability of heart and skeletal muscle development and is expected to have a higher gene dosage in DS^50^. Consistent with this observation, our RNA-Seq data showed an up-regulation of COL6A1 (FC = 1.25, pV = 1.11 × 10^−59^, Chr. 21), COL6A2 (FC = 1.79, pV = 5.34 × 10^−181^, Chr. 21), and the COL6A3 gene (FC = 1.36, pV = 0, Chr. 2). Additional collagen genes implicated in connective tissue development were located via the NCBI Gene database. Similar to Collagen VI gene expression, our DS mesodermal progenitors also exhibited a widespread Collagen II, III, XV, and XVI dysregulation across multiple chromosomes. More specifically, COL2A1 (FC = 1.42, pV = 2.82 × 10^−3^, Chr. 12), COL3A1 (FC = 1.96, pV = 0, Chr. 2), COL15A1 (FC = 0.50, pV = 8.54 × 10^−9^, Chr. 9), and COL16A1 (FC = 1.27, pV = 2.41 × 10^−35^, Chr. 1) gene expression showed statistically significant variation. Together these results suggest that, functionally, DS mesodermal progenitors will not perform as well as disomic mesodermal progenitors.

Our functional assay corroborated these results. The CFU assay demonstrated that even though DS iPSCs have an increased potential to generate mesenchymal colonies, when placed in semi-solid media, the progenitors failed to proliferate and formed significantly less colonies than the disomic control.

Cell cycle regulation was considered another possible cause for poor progenitor cell proliferation. USP16 is a histone de-ubiquitinating enzyme that regulates DNA damage response. Studies in mice demonstrated that over-expression of USP16 leads to failure in satellite cell expansion, which impairs muscle regeneration^51^. In combination with this, sphingosine-1-phosphate (S1P) complexes are additional cell cycle regulators implicated in mesodermal impairment. S1P is a pleiotropic lysophospholipid mediator that regulates cell proliferation, migration, survival, and differentiation. It acts though members of the G protein-coupled S1P receptors, such as S1P1, S1P2, and S1P3^52^. We identified a significant up- regulation of S1P2, which is widely expressed and regulates many different processes throughout the body^53^. A prominent example is the inhibition of vascular endothelial, smooth muscle, and tumor cell migration via activation of Rho and inhibition of Rac^54–59^. Furthermore, in mice, S1P2 has been found to strongly inhibit angiogenesis in endothelial and CD11b-positive bone marrow-derived cells^60^. The up-regulation of this receptor in mesodermal progenitors provides another explanation for the lower prevalence of solid tumors in DS individuals.

On the endothelial front, using STRING analysis, we identified that the most down- regulated processes are related to cellular response to stimuli, migration, and immune response (inflammation-based). To substantiate this finding, we evaluated the difference between VEGF response of DS endothelial progenitors compared to the disomic control. We observed that the expression of VEGF response was consistently lower in DS endothelial cells in comparison to disomic endothelial cells. Furthermore, DS cells had a down-regulated response to inflammatory TNF-α stimulus. Together, these results confirm our finding that DS endothelial cell functionally is impaired and potentially provides a mechanism for lower solid tumor development due to an inability to easily recruit endothelial cells.

Additional endothelial regulation stems from hypoxia inducible factor 1 (HIF-1). HIF-1 is a critical regulator of a wide array of physiological processes, including: angiogenesis, ECM remodeling, cytoskeletal rearrangements, and inflammation^61–64^. By regulating many cellular factors, this complex is capable of helping tumors to adjust to hypoxic conditions. Interestingly, the HIF-1 complex is down- regulated in DS endothelial cells. This finding is consistent with our functional analysis demonstrating a down-regulation of inflammatory response. Overall, the decreased expression of the HIF-1 complex would limit its capability to respond to tumor-induced signaling.

Furthermore, while examining elements of cellular characterization and functionality, we decided to pair our phenotypic observations with genotypic RNA-Seq data in order to develop a preliminary DS cancer profile. With regard to Chromosome 21 cancer-related gene expression, in both mesodermal progenitors (4C4-dMPs, 4C4-tMPs) and endothelial cells (DS-iECs, isoDS-iECs), we noted that the identified differentially expressed genes regulate a wide range of processes, including: immune response, hematopoietic development, DNA replication and repair, RNA splicing, connective tissue organization, migration, cellular metabolism, and solute transport. All of these processes are crucial aspects during cancer development. Due to the extra copy of Chromosome 21, it was not surprising that both trisomic mesodermal 4C4-tMPs and endothelial DS-iECs exhibited up-regulatory trends in gene expression. These data suggest that irrespective of cell lineage, these cell lines are significantly and similarly impacted by the genomic implications of T21.

In light of these results, we performed a genome-wide cancer-related gene expression level analysis to identify potential correlations. To substantiate our analysis, we surveyed the most mutated, non-Chromosome 21 genes across 10 cancer types. Although there was hardly any overlap between the gene lists (only 3 genes), both 4C4-tMPs and DS-iECs exhibited a down-regulatory expression trend in comparison to Chromosome 21 cancer-related genes. Such tendencies may imply that the DS cells are employing widespread genomic mechanisms to counter the significant up-regulation of Chromosome 21 genes. Furthermore, the non-Chr. 21 down-regulated, cancer-related genes had fold changes measuring 2–3 × greater than that of Chromosome 21 up-regulated genes. Considering all of these factors, perhaps such widespread genomic efforts are key contributors to lowering DS solid tumor prevalence.

miRNAs are also crucial components in the DS cancer profile. They are small non-coding RNAs that are involved in regulating gene expression. They participate in a variety of physiological processes, including carcinogenesis. Within our data set, we identified eight significantly expressed cancer-associated miRs, two of which were significantly down-regulated in both sets of isogenic stromal progenitors, miR-24-4 and miR-21 respectively. Recently, miR-24-2 has been proposed as a potential diagnostic marker for Esophageal Squamous Cell Carcinoma^65^. miR-21, on the other hand, is classified as an oncogene^66^ because it exerts an anti-apoptotic function in cancer cells^67^ as well as induces angiogenesis by activating Akt/ERK signaling pathways, which increase HIF-1α and VEGF expression^68^.

Another contributor to our cancer profile is the significant down-regulation of key ECM components. Collagen IV is a major component of the basement membrane (BM). It acts as a barrier that separates the epithelium from surrounding stroma^69^. While collagen was traditionally regarded as a passive barrier to resist tumor cells, recent studies show that it is actively involved in promoting tumor progression. More specifically, the loss of a critical component of type IV collagen chains can potentially increase the invasiveness of colorectal, and possibly other cancers^70^. Another study identified a compound that selectively targets Collagen IV and prevents Collagen IV expansion, which has been associated with chemotherapeutic resistance following the epithelial-to-mesenchymal transition ^71^. Collagen type IV crosslinking also contributes to ECM deposition, which drives malignant tumor progression. Furthermore, crosslinking between Collagen IV and Collagen I may contribute to the formation of the pre-metastatic niche in lungs^39^. In addition to collagen, over-expression of ECM-related genes such as Galectin-1^72^, Fibronectin^38^, and Heparan Sulfate^73^ in cancers suggests their involvement in tumor development and progression. The down-regulation of these ECM components in our DS model provides one mechanism for lower solid tumor prevalence: the creation of a reduced ECM-cytoskeletal dynamic that does not reflect the elevated migratory interactions associated with tumorigenesis.

While previous studies have evaluated DS cells in search of new gene therapy targets, there has always been a concern about an adequate sample representation due to the multiple genes involved, including those not located on Chromosome 21 and those indirectly affected. Another limitation is that mostly adult tissues were being analyzed as opposed to progenitor stages where altered developmental cues may be adequately evaluated. This study overcame the above limitations by analyzing stromal progenitor cells developed from two pairs of isogenic iPSC lines.

Based on the genetic signature and functional assays, our model of DS iPSC-derived mesodermal progenitors and endothelial cells provided novel insights into aberrant development of connective tissues and lower prevalence of solid tumor formation in DS individuals. We propose that the same processes that specify the basis of connective tissue impairment observed in DS patients can potentially serve a cancer-resistant role and hinder tumor progression and metastasis. We further established that, although some genes on Chromosome 21 are up-regulated, the genome-wide cancer-related genes are down-regulated. This result implies that widespread genomic mechanisms are compensating the significant up-regulation of Chromosome 21 genes, thereby providing another possible tumor-resistant mechanism via the down-regulation of cancer-related genes.

## Methods

### Differentiation, isolation, and cell culture expansion

Isogenic disomic/trisomic iPSCs (4C4-Disomic/4C4-Trisomic) were kindly provided by Dr. Stuart Orkin. H9 (WA09) was purchased from WiCell. Isogenic disomic iPSCs (isoDS-iPSCs), trisomic iPSCs (DS-iPSCs), and disomic SR2-iPSCs were derived as previously described in^12,13^. Prior to differentiation, the cells were maintained on Matrigel-coated culture dishes in mTeSR1 medium (STEMCELL Technologies). Differentiation was induced via addition of CHIR99021 (STEMCELL Technologies). Endothelial differentiation was also supplemented with vascular endothelial growth factor (VEGF_165_) (R&D Systems). On day 4, the differentiated ECs were isolated by immuno-selection of CD31^+^CD144^+^ cells via a magnetic column (Miltenyi Biotec). Following this, the SR2 and DSV-iECs were grown on fibronectin-coated (10 μg/mL) (BD Biosciences) plates and cultured in VascuLife EnGS medium (LifeLine) at 37 °C and 5% CO_2_. Mesodermal progenitors were isolated by immuno-selection of CD73 + cells and cultured in EBM 2 medium (Lonza).

### Flow cytometry analysis

To verify endothelial marker expression, the iPSC-derived endothelial cells were analyzed via flow cytometry. The cells were harvested with StemPro Accutase (ThermoFisher Scientific), washed with ice-cold FACS buffer (PBS + 1% FBS + 2 mM EDTA), and incubated with conjugated antibodies CD31 PE, CD34 FITC, VE-Cadherin APC (Miltenyi Biotech) or CD73 APC for 30 min at 4 °C. Following this, the cells were washed with a 0.5% BSA/PBS solution. Data collection was performed via the FACSCalibur (BD Biosciences) and analyzed via the FlowJo software (version 10.5.3).

### Blast colony forming unit (CFU) assay

The blast colony assay was performed in MethoCult H4100 media mixed with SFEM (Stem Cell Tech) and supplemented with Heparin, LiCl, Glutamax MTG, Ascorbic Acid (all from Sigma-Aldrich), ExCyte (Millipore), FGF2, VEGF (Peprotech) and BIT 9,500 Serum Substitute (Stem Cell Tech).

### Proliferation assay

30,000 control and trisomic endothelial cells were seeded per well onto fibronectin-coated (10 μg/mL) (BD Biosciences) 6-well plates. The cells were cultured in VascuLife EnGS medium (LifeLine) containing varying VEGF concentrations (0.5 ng/mL, 2 ng/mL, 20 ng/mL). When one of the cell lines reached confluence, all cells for a particular VEGF concentration were harvested with StemPro Accutase (ThermoFisher Scientific) and a cell count was performed.

### Migration assay

DS-iECs, H9-ECs, and SR2-iECs were plated in triplicate at a density of 75,000 cells per well of a fibronectin-coated (10 μg/mL) 12-well plate. The cells were cultured in VascuLife EnGS medium (LifeLine). Utilizing a 1000 μL pipet tip, a cell-free gap was created per well. The following were the average scratch area widths for each cell type: 708.18 μM for DS-iECs, 714.39 μM for H9-ECs, and 705 μM for SR2-iECs. Images were taken at times: T = 0 h (initial gap) and T = 17 h (time point when first cell line (SR2-iECs) reached confluency). ImageJ software was used for the area measurements (MRI Wound Healing Tool). Following this, the migration rate was calculated using 17hrs as the total time frame of the assay.

### RNA isolation

Total RNA was extracted with the RNeasy Mini Kit (Qiagen) via the instructions provided in the manufacturer’s protocol. RNA quality and concentration were assessed via the Nanodrop.

### Microarray analysis

RNA aliquotes were submitted to the University of Chicago Genomics Facility. RNA samples were reverse transcribed into cDNA, which was hybridized onto a HumanHT-12 v4BeadChip that was scanned by Illumina iScan. The acquired data was processed and normalized via the iScan Control software. Fold-change gene expression comparisons were obtained using the R Studio software (Bioconductor package).

### RNA sequencing analysis

Aliquots of RNA were submitted to Northwestern University’s NUSeq Core. The mRNA library was prepared and the samples were analyzed using HiSeq 4,000 Sequencing 50 bp, Single Reads. The obtained list of differentially expressed genes was further analyzed using MetaCore (Clarivate Analytics, version 20.1.1) and R Studio software (version 3.6.1). The gplots and pheatmap packages were incorporated into the R script.

## Supplementary information

Supplementary Information 1.
