# Supplementary material for: iPSC-derived progenitor stromal cells provide new insights into aberrant musculoskeletal development and resistance to cancer in down syndrome
Source: Sci Rep. 2020 Aug 6;10:13252. doi: 10.1038/s41598-020-69418-9 (PMC7414019; doi:10.1038/s41598-020-69418-9)
Supplement: Supplementary file 1 — Supplementary Information 1. [file 41598_2020_69418_MOESM1_ESM.pdf]

iPSC–Derived Progenitor Stromal Cells Provide New Insights into Aberrant Musculoskeletal Development and Resistance to Cancer in Down syndrome

Yekaterina Galat[1,7], Mariana Perepitchka[1,7], Irina Elcheva[1,6], Stephen Iannaccone[1], Philip M Iannaccone[1,2,3] and Vasiliy Galat[1,3,5]

[1]Developmental Biology Program, [2]Pediatrics, Stanley Manne Children's Research Institute, Ann & Robert H. Lurie Children's Hospital, [3]Pathology, [4]Robert H. Lurie Comprehensive Cancer Center, Northwestern University Feinberg School of Medicine, Chicago, IL, USA. [5]ARTEC Biotech Inc, Chicago, IL, USA. [6]Present Address: Pediatrics, Division of Hematology & Oncology, Penn State Hershey College of Medicine, Hershey, PA, USA.

[7]Co–first Author

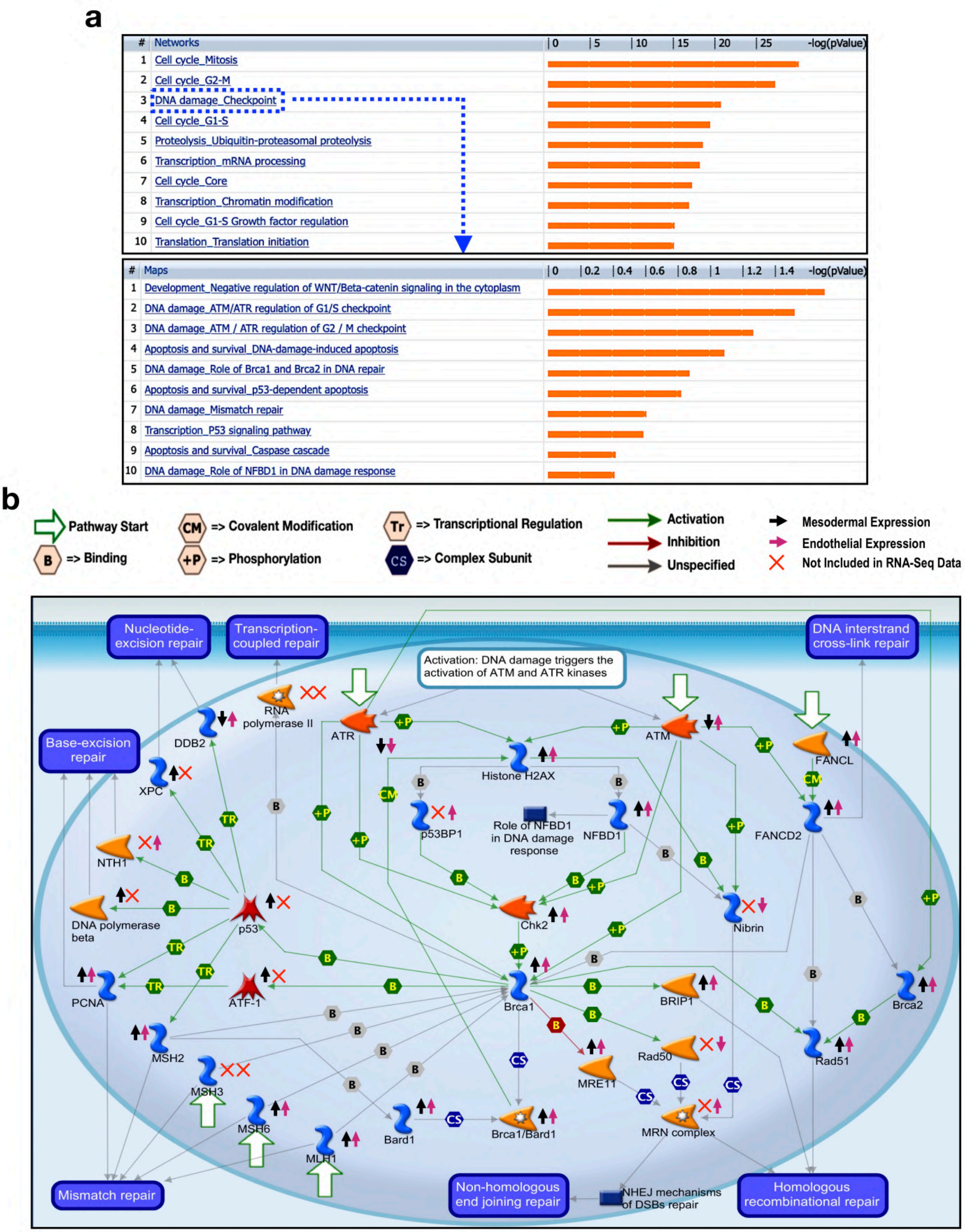

Supplemental Figure 1

(a). MetaCore enrichment analysis of mesodermal and endothelial RNA–Seq data showing the top 10 statistically significant functional networks and maps, which revolve around cell cycle phases and DNA damage response. **(b).** ATM and ATR kinase activation pathway depicting several down-stream DNA repair processes. The majority of the genes in this pathway are significantly up-regulated (pink and black arrows) in both mesodermal progenitors (4C4-dMPs, 4C4-tMPs) and endothelial cells (DS-iECs, isoDS-iECs); [p-Values < 0.001]. This pathway (Map ID: 427) was obtained from MetaCore's Pathway Map Creator application.

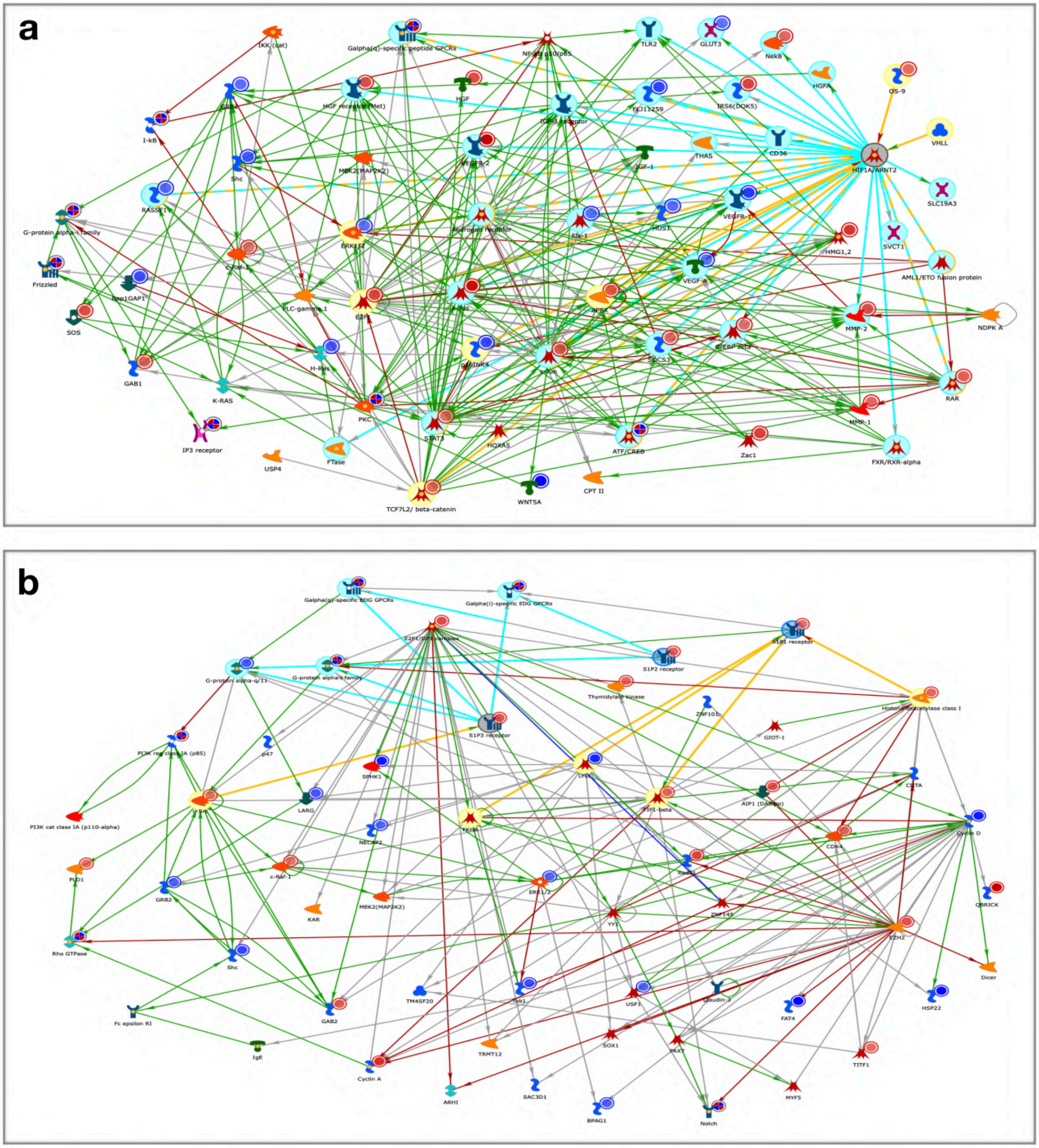

Supplemental Figure 2

(a).HIF-1 network generated via MetaCore for the endothelial (DS-iECs, isoDS-iECs) RNA-Seq dataset. The HIF-1 complex and its direct interactions have been highlighted. **(b).**S1P receptor complex network generated via MetaCore for the mesodermal progenitor (4C4-dMPs, 4C4-tMPs) RNA-Seq dataset. All of the genes shown in both networks have statistically significant expression levels; [p-Values < 0.001]. The green arrows symbolize activating interactions, the red arrows represent inhibition, and the grey arrows show non-specific interactions. The red circles represent up-regulated expression, and the blue circles symbolize down-regulated expression.

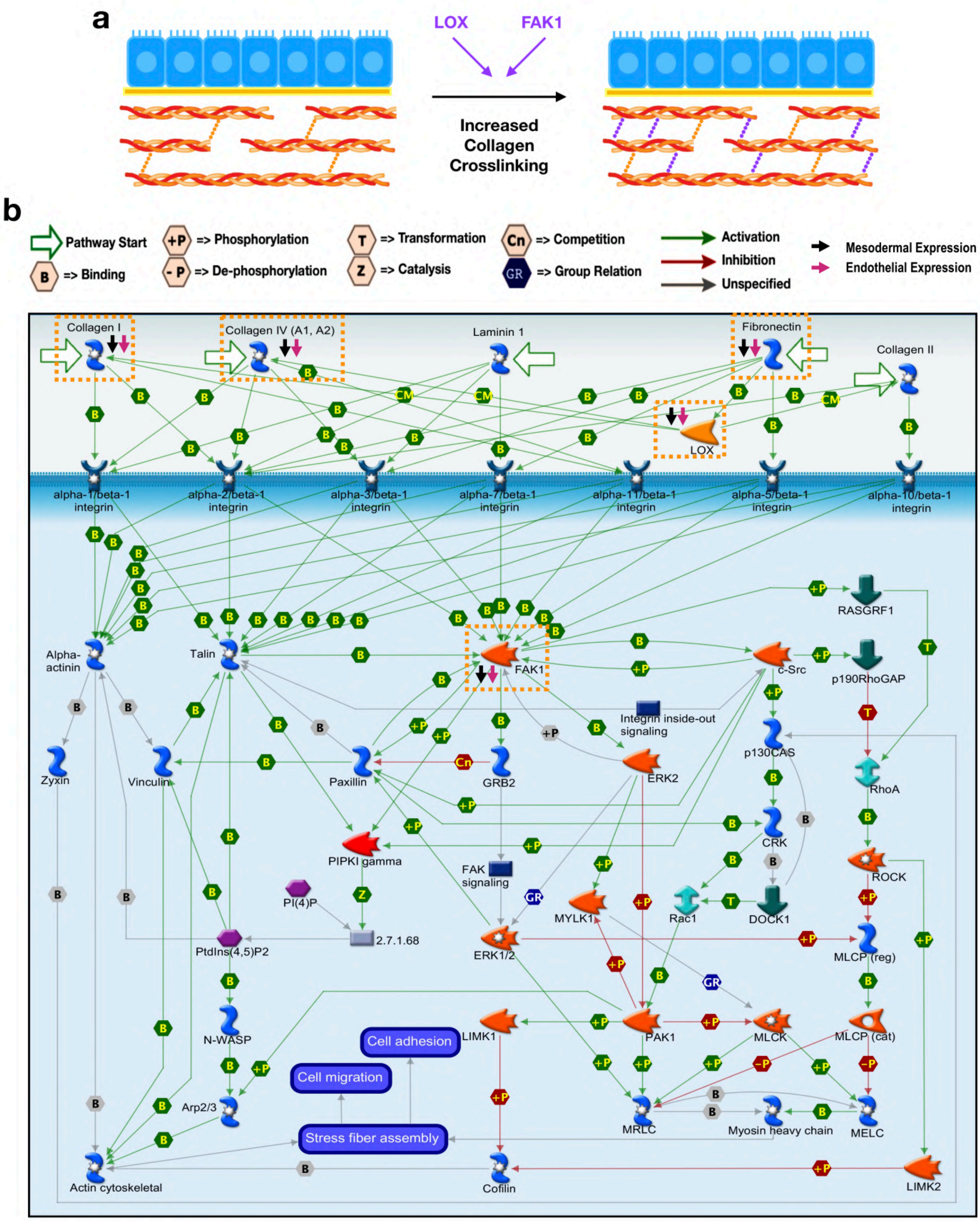

Supplemental Figure 3

(a). Schematic representation of LOX and FAK1 contributing toward increased collagen cross-linking, which creates a favorable environment for tumor progression. **(b).** Pathway depicting the interplay of Fibronectin, Collagen I, Collagen IV (A1 and A2 chains), FAK1, and LOX genes in ECM organization and cell migration. These genes are significantly down-regulated (pink and black arrows) in both mesodermal progenitors (4C4-dMPs, 4C4-tMPs) and endothelial cells (DS-iECs, isoDS-iECs); [p-Values < 0.001]. This pathway (Map ID: 450) was obtained from MetaCore's Pathway Map Creator application.
